# Vitamin B12 Deficiency, a Rare Cause of Isolated Thrombocytopenia in Adults

**DOI:** 10.7759/cureus.44162

**Published:** 2023-08-26

**Authors:** Muhammad Ahsan Naseer Khan, Usman Ghani, Salim Surani, Ayeman Aftab

**Affiliations:** 1 Internal Medicine, Mayo Hospital, Lahore, PAK; 2 Cardiology, Northwest General Hospital and Research Center, Peshawar, PAK; 3 Internal Medicine, Texas A&M University, College Station, USA; 4 Internal Medicine, Mahsa University, Kuala Lumpur, MYS

**Keywords:** vitamin b12, case report, mecobalamin, adults, isolated thrombocytopenia

## Abstract

Isolated thrombocytopenia in adults is a common clinical problem, often caused by various hematological disorders. However, vitamin B12 deficiency as a rare cause of isolated thrombocytopenia has been rarely reported in the medical literature. This case report aims to highlight the diagnostic challenges associated with atypical presentations of thrombocytopenia and emphasizes the importance of considering nutritional deficiencies, such as vitamin B12 deficiency, in the diagnostic workup. We report the case of a 38-year-old male who presented with generalized weakness, fatigue, and a history of bruises without trauma. Physical examination and laboratory investigations revealed thrombocytopenia (42 K/µL) with normal red blood cell morphology and no apparent abnormalities in other hematological parameters. Serum vitamin B12 levels were significantly diminished (128 pg/ml). The patient was treated with subcutaneous mecobalamin 1000 mcg supplementation, resulting in improvements in serum vitamin B12 levels (772 pg/ml) and platelet count (154 × 10^9^/L) values. This case highlights the importance of considering vitamin B12 deficiency as a potential cause of isolated thrombocytopenia in adults. The lack of hypersegmented neutrophils and characteristic signs of macrocytic anemia in the context of vitamin B12 deficiency emphasizes the necessity for a thorough investigation to rule out other possible causes. Hematological problems associated with thrombocytopenia caused by vitamin B12 deficiency can be treated early to resolve them and avoid complications.

## Introduction

To diagnose thrombocytopenia, a platelet count of less than 150 × 109/L is considered clinically significant; however, many consider a cutoff value of 100 × 109/L to be more acceptable. Rarely do patients with a platelet count over 50,000/mL exhibit symptoms [1]. Spontaneous bleeding is most common in patients with a platelet count below 20,000/mL. Common causes of thrombocytopenia include myelodysplasia and malignancies associated with chronic disseminated intravascular coagulation (DIC) or marrow suppression (leukemia, lymphoma, and solid tumors), paroxysmal nocturnal hemoglobinuria (PNH), thrombotic microangiopathy (TMA), thrombotic thrombocytopenic purpura (TTP), and hemolytic uremic syndrome (HUS). Other less common causes include aplastic anemia, inherited thrombocytopenia, and specific genetic disorders such as Wiskott-Aldrich syndrome, Fanconi syndrome, thrombocytopenia-absent radius syndrome, Bernard-Soulier syndrome, and May-Hegglin anomaly. Common causes of vitamin B12 deficiency include inadequate dietary intake [2], medications, and autoimmune and infectious gastrointestinal syndromes. Vitamin B12 is rarely seen in patients as a cause of isolated thrombocytopenia. Idiopathic thrombocytopenic purpura, chronic alcoholism, and liver cirrhosis have an increasing incidence in the United States due to increased alcohol consumption as one of the leading factors. According to extensive surveys conducted in the United States and the United Kingdom, roughly 6% of people over the age of 60 have low levels of vitamin B12 in their blood (plasma vitamin B12: 148 pmol/L), and this frequency increases with age. In later life, 20% or less have marginal status (plasma vitamin B12: 148-221 pmol/L) [3]. In the case of thrombocytopenia, 16.5% of people have thrombocytopenia, with 18.8% of men and 14.4% of women [4].

## Case presentation

A 38-year-old male presented to our services with generalized weakness, fatigue, and diarrhea. The patient had bruises without trauma on the upper and lower extremities in the past three to four years. The patient was normotensive, non-diabetic, and had no adverse drug reactions. Family history for any hereditary or chronic disease was non-significant. The patient denied experiencing chest pain, palpitations, dizziness, abdominal pain, nausea, vomiting, fever, and changes in appetite. The patient was non-alcoholic. The patient followed a non-vegan diet. During the examination, the patient was alert, awake, and oriented to time, place, and person. A complete physical and neurological examination, including gait, reflexes, vibration, and the Romberg test, revealed no abnormalities. A complete blood count revealed thrombocytopenia (hemoglobin level of 15.1 g/dL), hematocrit of 46.8%, red blood cell count of 6 million/mm3, white blood cell count of 6.96 K/µL, platelet count of 42 K/µL, mean platelet volume of 12.6 fL, and mean corpuscular volume (MCV) of 82 fL) as shown in Table 1, prompting further examination. 

**Table 1:** Laboratory data on admission

Labs	Value	Reference range
Complete Blood Count (CBC)		
White blood cell count (WBCs)	6.96 K/uL	4.8-10 K/uL
Red blood cell count (RBC)	6 million/mm3	4.5-5.5 million/mm3
Hemoglobin (Hb)	14.9 g/dL	14-18 g/dL
Hematocrit	44.9 %	42-52
Mean corpuscular volume (MCV)	82 fL	80-99
Platelets (PLT)	42 K/uL	(150-450) K/uL
Mean Platelet Volume (MPV)	12.6 fL	7.2-11 fL
Coagulation profile		
International Normalized Ratio (INR)	0.9	0.8-1.1
Platelet Time (PT)	10.2	1-13 seconds
Liver Function Tests (LFTs)		
Total Bilirubin	0.58 mg/dL	0.0-1 mg/dL
Alanine transaminase (ALT)	24 U/L	10-50 U/L
Aspartate transaminase (AST)	20 U/L	10-50 U/L
Kidney Function Tests (KFTs)		
Blood Urea Nitrogen (BUN)	10.37 mg/dL	6-20 mg/dL
Creatinine	0.87 mg/dL	0.7-1.2 mg/dL

Peripheral blood smear analysis exhibited a normocytic, normochromic picture of RBCs, indicating no apparent anomalies in erythrocyte morphology. The WBC count and morphology were within the reference range, and platelets were found to be reduced, but no clumping was observed. The reticulocyte count was 1.24%, indicating an appropriate bone marrow response [5]. The erythrocyte sedimentation rate (ESR) was found to be 5 mm/hr. The rheumatoid arthritis (RA) factor and anti-nuclear antibody (ANA) testing yielded negative results. Levels of serum folate (11.9 ng/ml), ferritin (133 ng/ml), and lactate dehydrogenase (LDH) (136 U/L) were also within normal ranges, excluding deficiencies and cellular damage as contributing factors. Notably, serum vitamin B12 levels were significantly diminished at 128 pg/ml, indicating a potential association with the observed thrombocytopenia. The laboratory data to investigate the possible cause of thrombocytopenia is presented in Table 2. 

**Table 2:** Laboratory data to investigate thrombocytopenia

Tests	Value	Reference range
Erythrocyte sedimentation rate (ESR)	5 mm/1st hr	0-15 mm/1^st^ hr
Reticulocyte Count	1.24 %	0.5-2.5 %
Ferritin	133 ng/ml	28-365 ng/ml
Vitamin B12	128 pg/ml	211-911 pg/ml
Folate	11.9 ng/ml	3.0-17.0 ng/ml
Anti-nuclear antibody (ANA)	Negative	
Rheumatoid factor	7 IU/ml	<14 IU/ml
Lactate dehydrogenase (LDH)	136 U/L	135-225 U/L

The patient was started on the subcutaneous administration of mecobalamin. A dosage of 1000 mcg of mecobalamin was recommended daily for 10 days, followed by a transition to weekly administration for the subsequent three weeks. The patient's response to the prescribed treatment regimen was assessed through subsequent CBC analysis and measurement of serum vitamin B12 levels. The results revealed notable improvements in both parameters. The post-treatment serum vitamin B12 level was measured at 772 pg/ml, demonstrating a significant increase from the baseline value of 128 pg/ml. This significant spike suggests that vitamin B12 reserves have been well restored. The patient's platelet count also showed a noticeable improvement concurrently. The post-treatment CBC analysis revealed a platelet count of 154 × 109/L and a mean platelet volume of 11 fL, indicating a substantial improvement from the initial thrombocytopenic state. In addition, significant clinical improvement of symptoms, including fatigue and generalized weakness, was also observed [6]. The patient was advised to continue subcutaneous mecobalamin injections weekly and take an oral vitamin B1 (thiamine disulfide) 100 mg, vitamin B6 (pyridoxine hydrochloride) 200 mg, and vitamin B12 (cyanocobalamin) 200 mcg tablet once a day [7]. The patient was followed up on this treatment regimen for six months. Both vitamin B12 levels and platelet count on follow-up are presented in Table 3. Increased platelet count and blood vitamin B12 levels were observed, indicating a positive treatment response and corroborating the link between thrombocytopenia and vitamin B12 insufficiency.

**Table 3:** Laboratory data on follow-ups

Duration	Vitamin B12 levels	Platelet count
After one month	772 pg/ml	154 × 10^9^/L
After three months	569 pg/ml	170 × 10^9^/L
After six months	549 pg/ml	166 × 10^9^/L

## Discussion

Hematological abnormalities are frequently observed in vitamin B12 deficiency [8]. Macrocytic anemia, characterized by megaloblasts with an enlarged MCV, is frequently caused by vitamin B12 deficiency. Pancytopenia and hypersegmented neutrophils may be seen when the peripheral blood smear is examined. The solitary thrombocytopenia that occurs in the setting of vitamin B12 deficiency without the usual signs of macrocytic anemia and hypersegmented neutrophils is unusual and calls for further investigations. The absence of other hematological abnormalities and the subsequent correction of platelet levels through vitamin B12 supplementation on regular follow-ups effectively rule out immune thrombocytopenic purpura and other etiologies. In addition, there have been rare reports in the medical literature documenting such atypical presentations [9,10]. Vitamin B12 serves as a cofactor for methionine synthase and methylmalonyl-CoA mutase involved in the synthesis phase of platelets [11]. Methionine synthase participates in the conversion of homocysteine to methionine. Methionine is required for the synthesis of proteins, including those involved in platelet production. Methylmalonyl-CoA mutase is involved in the conversion of methylmalonyl-CoA to succinyl-CoA. Vitamin B12 deficiency can impair platelet production, including DNA replication and cell division required for cell maturation. Megakaryocytes do not develop and mature adequately, which reduces the production of platelets. The release of platelets from megakaryocytes may be impacted by inadequate energy generation because of decreased methylmalonyl-CoA mutase activity. This can further contribute to a reduced platelet count. Thus, this case highlights the diagnostic challenges that can arise when encountering atypical manifestations of thrombocytopenia. The absence of characteristic findings, such as abnormal RBC morphology or positive autoimmune serology, highlights the importance of considering alternative etiologies, including vitamin B12 deficiency, in the diagnostic workup of thrombocytopenia. This case serves as a reminder for healthcare providers to consider vitamin B12 deficiency as a potential cause of thrombocytopenia [10], particularly in the absence of other identifiable etiologies. 

## Conclusions

Early diagnosis and treatment of vitamin B12 deficiency-related thrombocytopenia can lead to the correction of hematological abnormalities and the prevention of further complications. Rare presentations, such as in this case, add to the body of currently known scientific knowledge by increasing our comprehension of the various causes of thrombocytopenia. We recommend more studies be done to elucidate the underlying mechanisms linking vitamin B12 deficiency and thrombocytopenia, improve diagnostic procedures, and investigate the best therapeutic options.
